# The effect of macromolecular crowders on deposition of extracellular matrix in astrocyte cultures

**DOI:** 10.1007/s00441-025-03980-4

**Published:** 2025-05-19

**Authors:** Sorour Nemati, Michelle Kilcoyne, Dimitrios Zeugolis, Siobhan S. McMahon

**Affiliations:** 1 Anatomy, School of Medicine, University of Galway, Galway, Ireland; 2 CÚRAM Research Ireland Centre for Medical Devices, University of Galway, Galway, Ireland; 3 Carbohydrate Signalling Group, Infectious Disease Section, School of Biological and Chemical Sciences, University of Galway, Galway, Ireland; 4 Regenerative, Modular & Developmental Engineering Laboratory (REMODEL), Charles Institute of Dermatology, Conway Institute of Biomolecular & Biomedical Research and School of Mechanical & Materials Engineering, University College Dublin (UCD), Dublin, Ireland; 5 Galway Neuroscience Centre, University of Galway, Galway, Ireland

**Keywords:** Macromolecular crowding, Extracellular matrix, Glial scar, Fibrotic scar, Astrocytes, Spinal cord injury

## Abstract

Macromolecular crowding (MMC) is a biophysical phenomenon that has proven effective in enhancing extracellular matrix (ECM) deposition in vitro. However, MMCs potential in neuroglial cell cultures remains underexplored. This study investigates the effects of three distinct MMC agents [carrageenan (CR), dextran sulphate (DxS) and FicollⓇ cocktail (FC)] on ECM deposition and cell behaviour of Neu7 and primary astrocytes. While the viability and metabolic activity of Neu7 astrocytes were unaffected by any of the crowding agents, primary astrocytes exhibited a significant decrease in viability and metabolic activity in the presence of CR and DxS. The addition of CR, DxS, and FC resulted in a significant increase in deposition of fibronectin, collagen IV, collagen I, GFAP and CS56 in Neu7 astrocytes. In primary astrocytes, FC significantly enhanced the expression of astrocytic markers and increased the deposition of ECM proteins, including fibronectin and collagen IV. This study highlights the advantages of using FC as a MMC agent for enhancing ECM deposition in astrocytes. The method demonstrates potential for developing fast and more physiologically relevant in vitro models and improving drug screening processes for future studies. The observed benefits underscore the utility of FC in creating advanced cellular models that better mimic the native neural environment.

**Supplementary Information:**

The online version contains supplementary material available at 10.1007/s00441-025-03980-4.

## Introduction

Macromolecular crowding (MMC), a phenomenon characterized by the dense packing of macromolecules within cellular and extracellular spaces, plays a critical role in shaping the biochemical and biophysical environment of cells (Zeiger et al. 2012; Raghunath and Zeugolis 2021). In physiological conditions, cells are surrounded by a crowded molecular milieu composed of proteins, nucleic acids, polysaccharides and other macromolecules. This crowding effect influences various cellular processes, including protein folding, molecular interactions, enzymatic reactions and signal transduction pathways (Alfano et al. 2024). The crowded environment imposes steric hindrance and alters the diffusion properties of molecules, leading to changes in reaction rates, equilibrium constants and macromolecular conformations (Silverstein and Slade 2019). MMC reduces the available solvent volume due to high concentrations of macromolecules, leading to the excluded volume effect, which increases the concentration and chemical activity of macromolecules (Lareu et al. 2007). In recent years, researchers have recognized the significance of MMC in cellular physiology and pathology, particularly in the context of extracellular matrix (ECM) protein deposition and scar formation (De Pieri et al. 2020; Marinkovic et al. 2021; Ramalingam et al. 2023; Zeugolis 2021)). This accelerated deposition of ECM proteins, such as collagen, fibronectin, and laminin, is crucial for the formation of tissue scaffolds and the maintenance of tissue integrity (Martínez-Vidal et al. 2023).

Given the profound influence of MMC on cellular behaviour and tissue function, researchers have increasingly utilized MMCs in the development of in vitro models to better mimic the physiological and pathological conditions observed in vivo (Fan et al. 2020; Cavanzo et al. 2021; Coentro et al. 2021). These in vitro models employ synthetic polymers, polysaccharides, proteins, and other crowding agents to recreate the in vivo crowded molecular environment and study cellular processes in a more physiologically relevant context (Zeugolis 2021). In this study, three MMCs were utilised, carrageenan (CR), dextran sulphate (DxS) and FicollⓇ cocktail (FC). CR is a sulfated polysaccharide derived from red seaweed, often used as a thickening or gelling agent in food and pharmaceutical industries (Du et al. 2023; Graceffa and Zeugolis 2019). DxS is a sulfated polysaccharide composed of glucose residues, commonly employed in biomedical research for its anticoagulant properties and ability to mimic heparan sulfate in vitro (Ghosh et al. 2020; Shahid et al. 2017). FC is a synthetic and highly branched co-polymer of sucrose and epichlorohydrin widely used as a crowding agent in biological and biochemical studies due to its inert properties and ability to mimic the crowded extracellular environment (De Pieri et al. 2020). A FC cocktail (70 and 400 kDa) has also demonstrated potential, as mixed crowding, by generating artificial polydispersity, can more effectively exclude volume compared to their component single-domain counterparts (Gaspar et al. 2019).

Scar formation, particularly following spinal cord injury (SCI), represents a significant challenge in regenerative medicine (Quadri et al. 2020). SCI triggers a cascade of cellular and molecular events that culminate in the formation of glial and fibrotic scars, which contribute to the persistent functional deficits observed in patients (Li et al. 2021; Silver and Miller 2004). Glial scars, primarily composed of reactive astrocytes, form at the site of injury and are characterized by hypertrophic astrocytic processes and the upregulation of intermediate filament proteins such as glial fibrillary acidic protein (GFAP) (Wichmann et al. 2023). These scar-forming astrocytes, while initially serving a protective role by creating a barrier to limit the spread of inflammation and cell death, ultimately contribute to the inhibition of axonal regeneration and functional recovery through the secretion of inhibitory molecules such as chondroitin sulfate proteoglycans (CSPGs), tenascin-C, and the formation of a dense ECM network (Yang et al. 2020; Bijelić et al. 2021; Zhang et al. 2022a, b). In addition to glial scarring, SCI also induces the formation of fibrotic scars, characterized by the excessive deposition of ECM proteins such as collagen, fibronectin, and laminin (D'Ambrosi and Apolloni 2020). Fibrotic scars are predominantly formed by fibroblasts, which infiltrate the injury site and deposit collagen-rich ECM, leading to tissue fibrosis and the formation of a mechanical barrier that impedes axonal regeneration and tissue remodelling (Ayazi et al. 2022). The presence of fibrotic scar tissue further exacerbates the inhibitory environment in the injured spinal cord and contributes to the formation of a non-permissive substrate for axonal growth (Jin et al. 2022). Early fibrosis is an important therapeutic target for improving outcomes after SCI, highlighting its potential role in the repair process (Bradbury and Burnside 2019). The interplay between glial and fibrotic scar components, along with the dynamic changes in the ECM composition and organization, creates a complex microenvironment that presents significant challenges for tissue repair and regeneration following SCI (Hu et al. 2023). Thus, understanding the cellular and molecular mechanisms underlying glial and fibrotic scar formation is crucial for developing effective therapeutic strategies to promote tissue repair and functional recovery in SCI patients (Orr and Gensel. 2018).

In this study, we investigated the effects of CR, DxS and FC on the Neu7 astrocyte cell line and primary astrocytes. Neu7 astrocyte cells are useful within in vitro models of glial scarring as they represent astrocytes that are inhibitory and produce CSPGs (Abu-Rub et al. 2016; Fidler et al. 1999; Kilcoyne et al. 2012). Understanding how different MMCs modulate glial and fibrotic scar formation in vitro can provide insights into the mechanisms underlying scar formation and potentially provide a model system for accelerated ECM deposition to identify novel therapeutic targets for SCI. By assessing the impact of MMCs on astrocytes, we aimed to elucidate their effects on cellular behaviour and ECM deposition.

## Results and discussion

### Effect of MMCs on growth of astrocytes

**Fig. 1:**
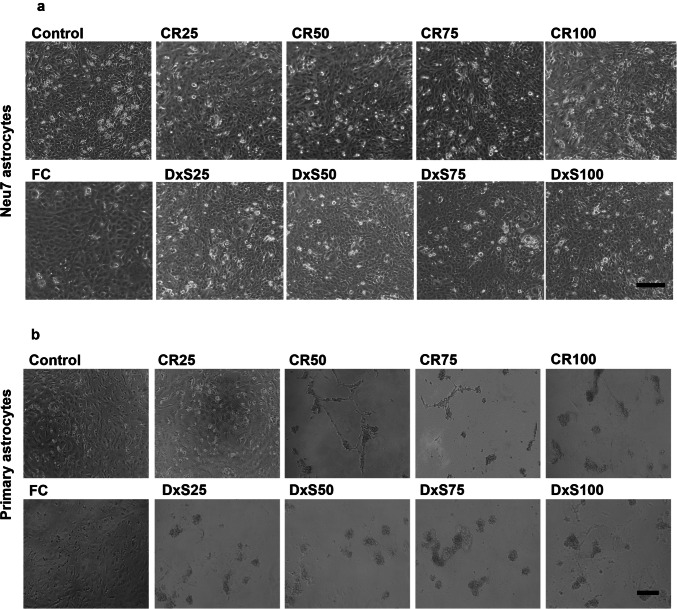
Cell morphology after treatment with MMCs. Phase contrast photomicrographs of Neu7 (**a**) and primary (**b**) astrocytes at 5 days without MMCs (Control) and with the MMCs FC, CR and DxS. Four concentrations of CR and DxS (25, 50, 75, 100 μg/ml) and FC (Ficoll 70 kDa (37.5 mg/ml) and Ficoll 400 kDa (400 mg/ml)) were used. Scale bars = 150 µm

The growth and morphology of astrocytes were examined without MMC treatment (control) and with treatment of the MMC agents CR, DxS and FC. The phase contrast images of Neu7 astrocyte morphology indicated that these cells were not affected by culture with FC at a single concentration or with different concentrations of CR and DxS at day 5 (Fig. 1a). The morphology of primary astrocytes did not change in the presence of FC compared to the control (Fig. 1b). However, the addition of CR and DxS at all concentrations affected the cell morphology of primary astrocytes and cells aggregated (Fig. 1b). Although CR did not completely eliminate the cells, the cells did show noticeable differences compared to the control group, with increased brightness around the edges of the cells, indicating signs of stress or early damage. The different behaviours of Neu7 astrocytes and primary astrocytes in the presence of CR, DxS and FC is suggested to be attributed to the charge of these MMCs. CR and DxS carry negative charge, whereas FC is neutral (De Pieri et al. 2020; Gaspar et al. 2019). Neu7 astrocytes remained relatively unaffected by CR, DxS and FC, suggesting that they may be more resilient to changes in the ionic environment or the presence of charged MMCs. However primary astrocytes, although unaffected by FC, showed significant morphological changes, including aggregation and detachment, in response to CR and DxS. This suggests that the negative charge of CR and DxS may play a significant role in affecting primary astrocyte morphology. The negative charge on CR and DxS likely created electrostatic repulsion or attraction with components of the cell membrane or ECM proteins in primary astrocytes, disrupting normal cell interactions and leading to aggregation and detachment. These findings are not surprising as cell lines (such as the Neu7 astrocyte cell line) are characteristically more robust than primary cells.

### Analysis of astrocyte cell health of after treatment with MMCs

**Fig. 2:**
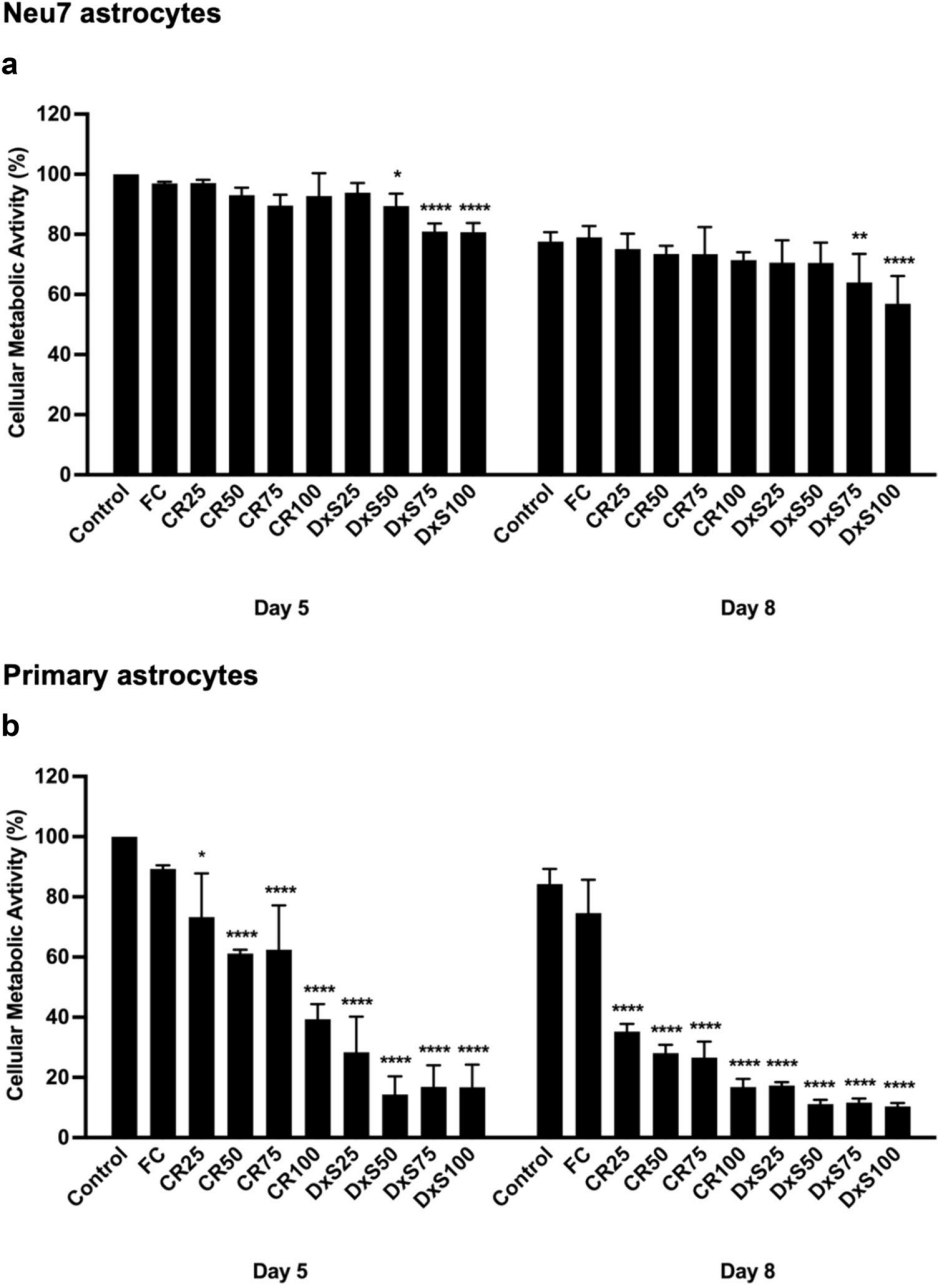
Metabolic activity of Neu7 and primary astrocytes after treatment with MMCs. The metabolic activity of Neu7 cells (**a**) and primary astrocytes (**b**) grown for 5 and 8 days without MMCs (Control) and with the MMCs FC, CR and DxS. Four concentrations of CR and DxS (25, 50, 75, 100 μg/ml) and FC (Ficoll 70 kDa (37.5 mg/ml) and Ficoll 400 kDa (400 mg/ml)) were used. Mean ± SD. * p = 0.04, ** p < 0.0045, **** p < 0.0001 compared to control. n = 3. Tukey’s posthoc test using one-way ANOVA

Both metabolic activity and cell viability were examined after treatment of astrocytes with CR, DxS and FC. The metabolic activity analysis of Neu7 astrocytes revealed that the Neu7 astrocytes treated with FC and different concentrations of CR did not exhibit significant deviations compared to the control group at day 5 (Fig. 2a). This observation indicates that CR and FC had no discernible negative impact on cellular metabolic activity of these cells. However, as the concentration of DxS increased to 50, 75, and 100 μg/ml, the metabolic activity significantly decreased compared to the control group at 5 days. At day 8, the Neu7 astrocytes detached, and the metabolic activity of all groups, including the control group, decreased. This decrease in metabolic activity in Neu7 astrocytes at day 8 can be attributed to the rapid growth rate of the Neu7 astrocyte cell line. In our experimental design, both cell control groups remained viable and metabolically active despite the high cell density, suggesting that over confluency alone was not the main contributor to cell stress or death. Rather, the observed effects, particularly the reduction in metabolic activity were more prominent in cells treated with the negatively charged MMCs. Primary astrocytes showed no significant change in metabolic activity when grown for 5 or 8 days with FC (Fig. 2b). However the metabolic activity of primary astrocytes decreased significantly compared to control cells when the astrocytes were cultured with DxS and CR at days 5 and 8.

**Fig. 3:**
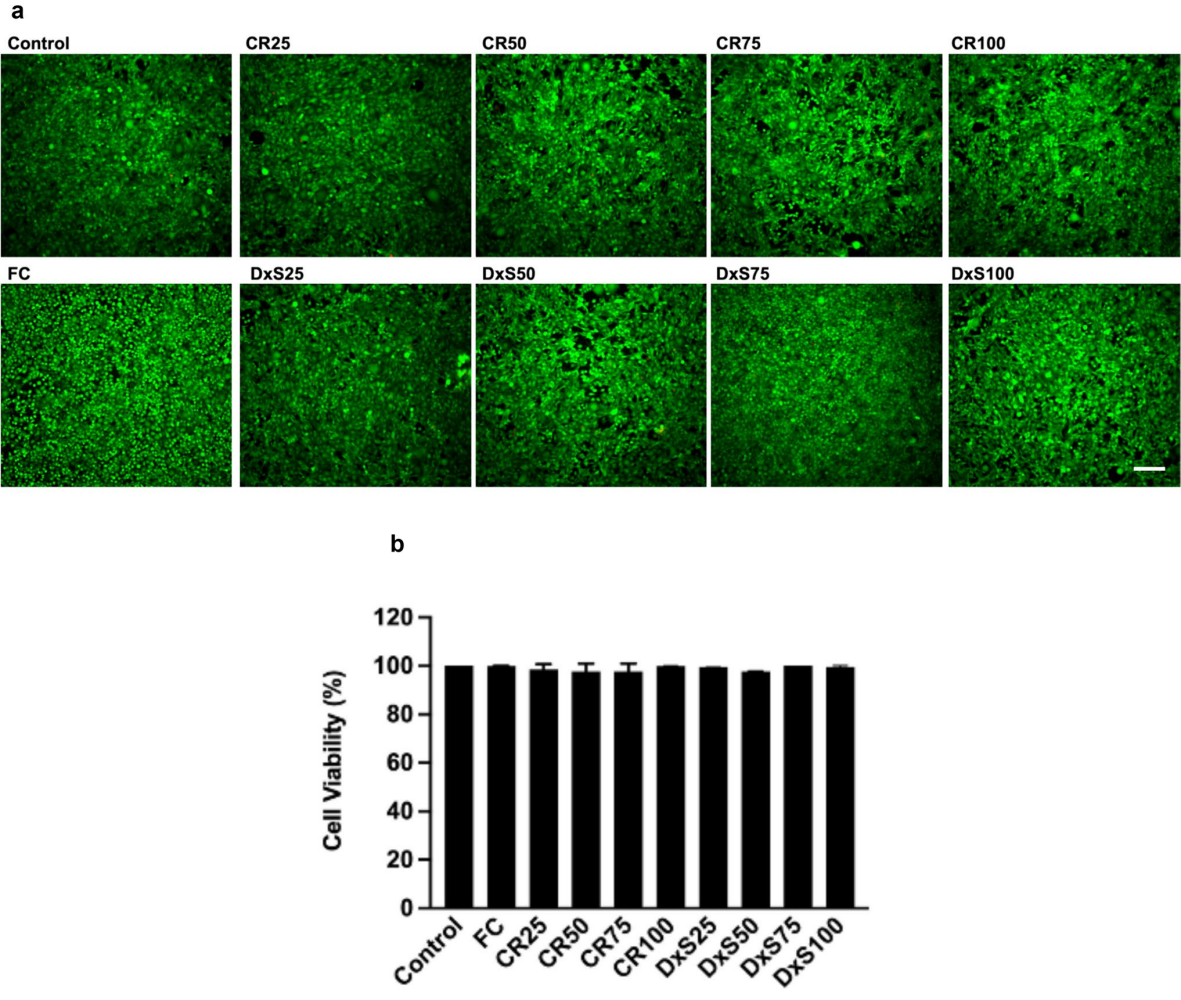
Cell viability of Neu7 astrocytes after treatment with MMCs. Neu7 cells grown for 5 days without MMCs (Control) and with the MMCs FC, CR and DxS. Four concentrations of CR and DxS (25, 50, 75, 100 μg/ml) and FC (Ficoll 70 kDa (37.5 mg/ml) and Ficoll 400 kDa (400 mg/ml)) were used. Representative photomicrographs show live cells (green) and dead cells (red) in each group (**a**). Bar chart shows quantitative analysis expressed in percentage of live cells (**b**). Scale bars = 200 µm. Mean ± SD. n = 3

**Fig. 4:**
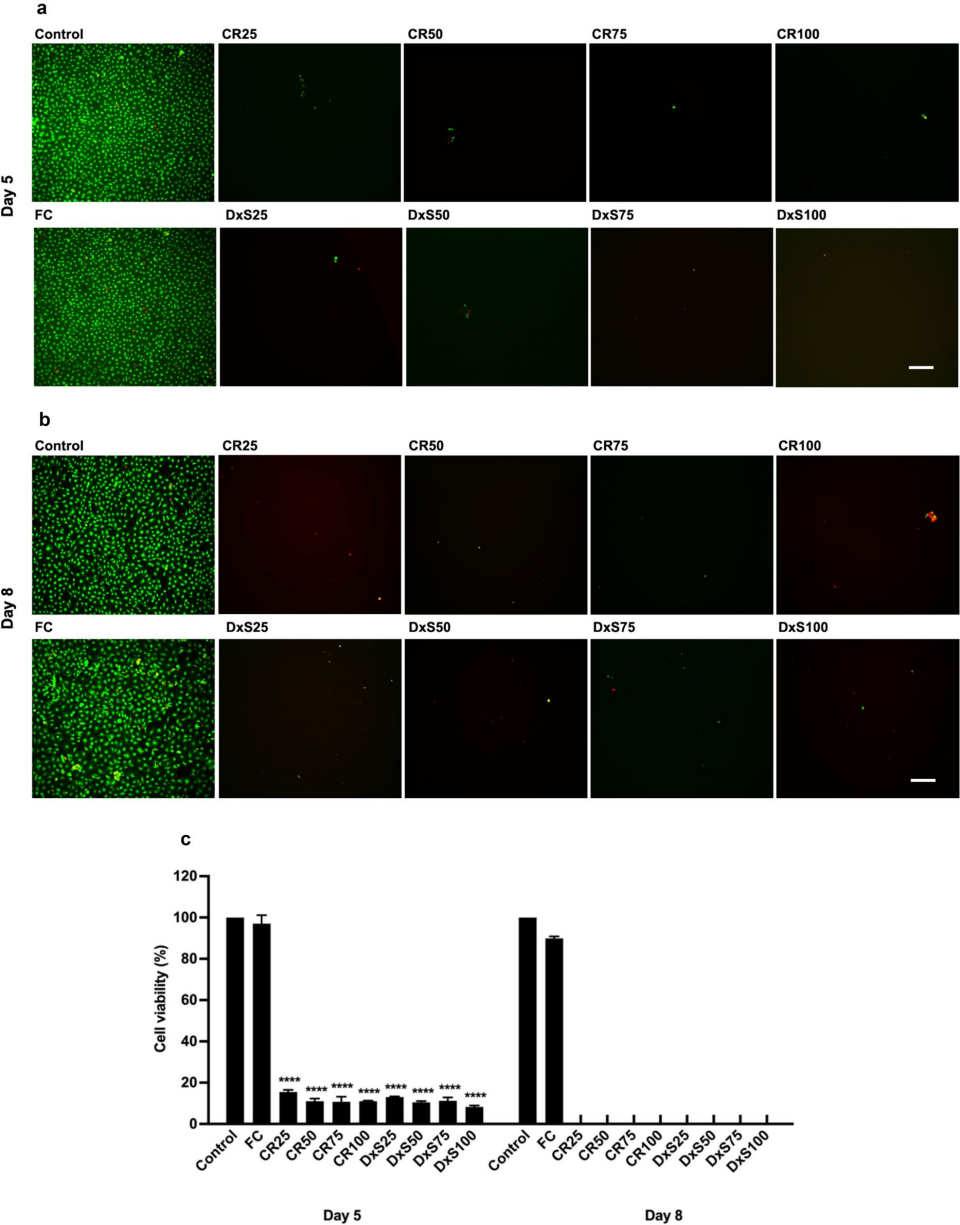
Cell viability of primary astrocytes after treatment with MMCs. Representative photomicrographs show live (green) and dead (red) primary astrocytes at day 5 (**a**) and day 8 (**b**) without MMCs (Control) and with the MMCs FC, CR and DxS. Four concentrations of CR and DxS (25, 50, 75, 100 μg/ml) and FC (Ficoll 70 kDa (37.5 mg/ml) and Ficoll 400 kDa (400 mg/ml)) were used. Bar chart shows quantitative analysis expressed in percentage of live cells (**c**). Scale bars = 200 µm. n = 3. Mean ± SD. **** p < 0.0001 compared to control. Tukey’s posthoc test using one-way ANOVA

Cell viability of Neu7 astrocytes was not altered after adding different MMCs at day 5 (Fig. 3a & b). Cell viability for the Neu7 astrocyte cell line was not assessed at day 8 due to cell detachment. The cell viability of primary astrocytes significantly decreased when DxS and CR were added to the culture media after day 5, and by day 8, the primary astrocytes had detached from the culture dish (Fig. 4). In contrast, the cell viability of primary astrocytes in the presence of FC remained unchanged compared to the control group at both time points (Fig. 4c). These findings indicate that Neu7 astrocytes maintained metabolic activity and cell viability in the presence of CR and FC, and low concentrations of DxS after 5 days. Primary astrocytes, however, were highly sensitive to both CR and DxS at all concentrations tested, leading to significant decreases in metabolic activity and viability, with FC having no negative impact. This suggests that CR and DxS affecting primary astrocytes more profoundly than Neu7 astrocytes.

Previously, it was found that various concentrations of CR, FC and DxS did not significantly impact cell morphology, viability, or metabolic activity in human adult dermal fibroblasts (Gaspar et al. 2019). A study using human adipose-derived stem cells (hADSCs) cultured with CR indicated that the addition of CR did not affect cell morphology or viability, suggesting that CR is a viable MMC candidate for use in hADSC cultures (De Pieri et al. 2020). Rampin et al. (2024) evaluated the fundamental function of human tenocytes in the presence of CR and FC. By day 7, the latest time point examined, neither CR nor FC had a significant impact on metabolic activity or cell viability. Our results highlight the influence of MMC agents on biological interactions in vitro, providing insights into how MMC can affect cellular behaviour.

### ECM deposition in MMC-treated astrocytes

Based on the metabolic activity and cell viability results, subsequent experiments were carried out for 5 days as Neu7 astrocyte cell lines grew very quickly and detached from the culture dish surface by day 8. A concentration of 75 μg/ml for CR (CR75) and DxS (DxS75) was selected as optimal for ECM deposition in Neu7 astrocytes, based on their biocompatibility and higher collagen I deposition (Supplementary Figure S1). Due to the significant decrease in metabolic activity and cell viability of primary astrocytes in the presence of DxS and CR, FC was exclusively chosen for subsequent analysis of ECM deposition in primary astrocytes.

**Fig. 5:**
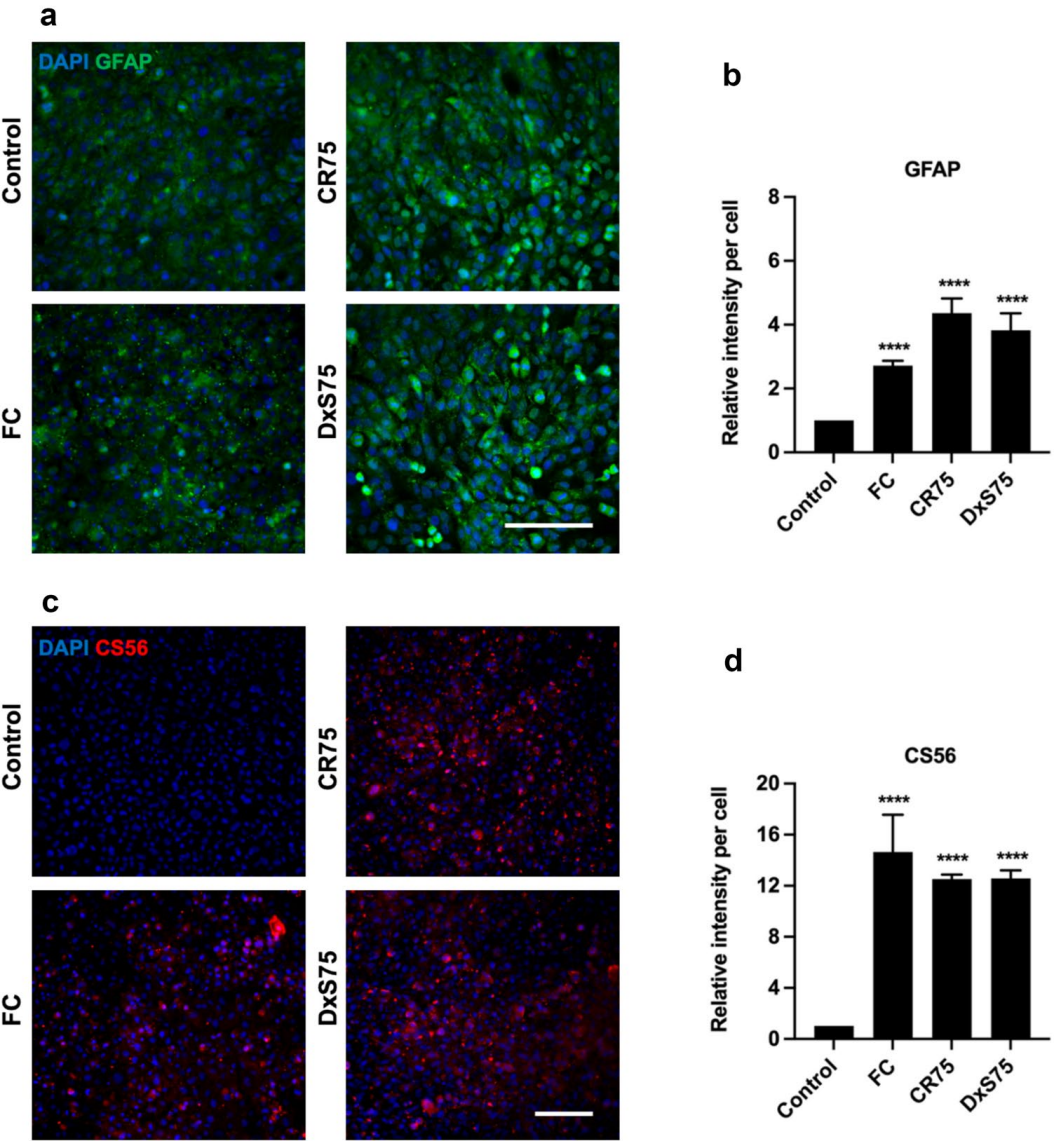
Effect of MMCs on glial scar proteins in Neu7 astrocyte cultures. Representative immunofluorescent photomicrographs show GFAP (**a**) and CS56 (**c**) expression in Neu7 astrocytes without MMC (control) and with the MMCs FC, CR75 and DxS75. Bar charts show the relative fluorescence intensity per cell analysis of GFAP (**b**) and CS56 (**d**). Scale bars = 100 µm. n = 3. Mean ± SD. **** p < 0.0001 compared to control. Tukey’s posthoc test using one-way ANOVA

**Fig. 6:**
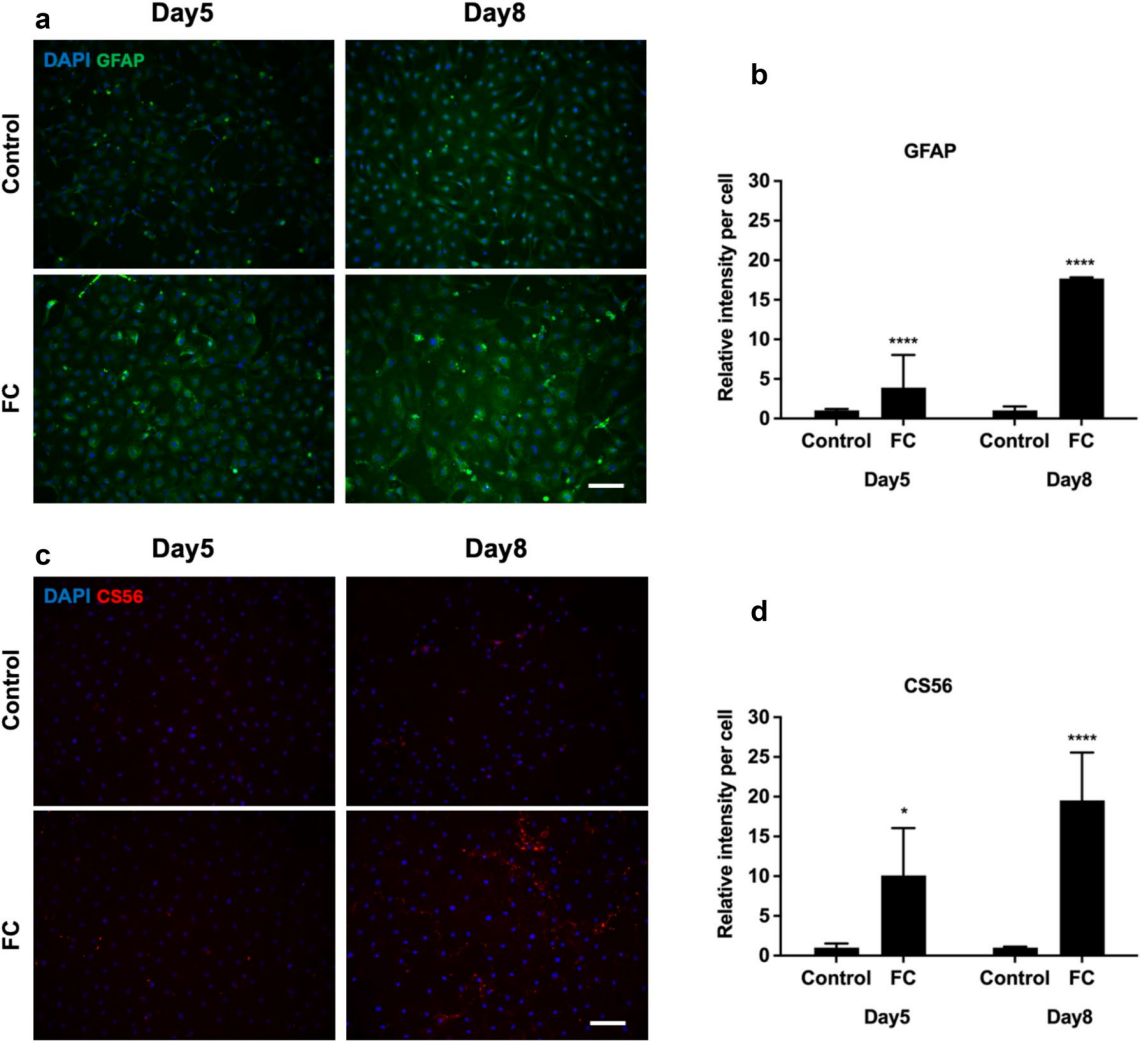
Effect of FC on glial scar proteins in primary astrocyte cultures. Representative immunofluorescent photomicrographs show GFAP (**a**) and CS56 (**c**) expression in primary astrocytes on day 5 and day 8 without FC (Control) and with the MMC FC. Bar charts show the relative fluorescence intensity per cell analysis of GFAP (**b**) and CS56 (**d**). Scale bars = 100 µm. n = 3. Mean ± SD. * p = 0.02 **** p < 0.0001 compared to control. Tukey’s posthoc test using one-way ANOVA

Immunocytochemical markers of the glial and fibrotic scar were examined following treatment of Neu7 and primary astrocytes with MMCs. In the glial scar astrocytes become reactive and over-express the intermediate filament protein GFAP (Pekny and Nilsson 2005). In addition cells within the glial scar express inhibitory CSPGs (Asher et al. 2001), which were quantified in this study using anti-CS56 antibody (CS56). The astrocytic marker GFAP (Fig. 5a, b) and the CSPG marker CS56 (Fig. 5c, d) were significantly increased in Neu7 astrocytes when cultured with CR75, DxS75, and FC on day 5 compared to control cells. Primary astrocytes after treatment with FC also showed significantly increased GFAP (Fig. 6a, b) and CS56 (Fig. 6c, d) on both days 5 and 8.

Neu7 astrocytes are a cell line engineered to overexpress CSPGs and normally exhibit elevated levels of CSPGs, even without injury (Fidler et al. 1999). This ability to produce CSPGs makes Neu7 cells particularly valuable for studying CSPG effects on glial scarring (Tuinstra et al. 2013). In contrast, primary astrocytes typically do not overexpress CSPGs, unless subjected to injury or stress (Hart and Karimi-Abdolrezaee 2021). However, in this study we showed that the addition of MMC increased CSPG levels in both Neu7 and primary astrocytes rapidly, even without applying injury to the cells.

In our in vitro model of glial scarring, both Neu7 astrocytes and primary astrocytes exhibit characteristics similar to those observed in in vivo models (Okada et al. 2018), particularly in their overexpression of GFAP (Zhang et al. 2017; Yu et al. 2021) and increased levels of CSPGs (Alizadeh et al. 2021; Hussein et al. 2020; Vangansewinkel et al. 2019). This resemblance of our MMC model to in vivo injury suggests that our model successfully mimics the reactive astrocyte behaviour seen in glial scar formation, where astrocytes upregulate GFAP and elevate CSPGs levels, which are hallmarks of astrocytic reactivity and glial formation in the spinal cord. The formation of the glial scar is one of the main limitations for repair following SCI. It has been demonstrated that glial scar formation is regulated by environmental cues, such as fibrotic ECM proteins (Okada et al. 2018). The ability of this MMC model system to include both glial and fibrotic scar markers makes it a unique model for assessing scar in an in vitro setting. This model also promotes reduction, replacement and refinement (3Rs) of animal use in SCI models (Slovinska et al. 2016).

**Fig. 7:**
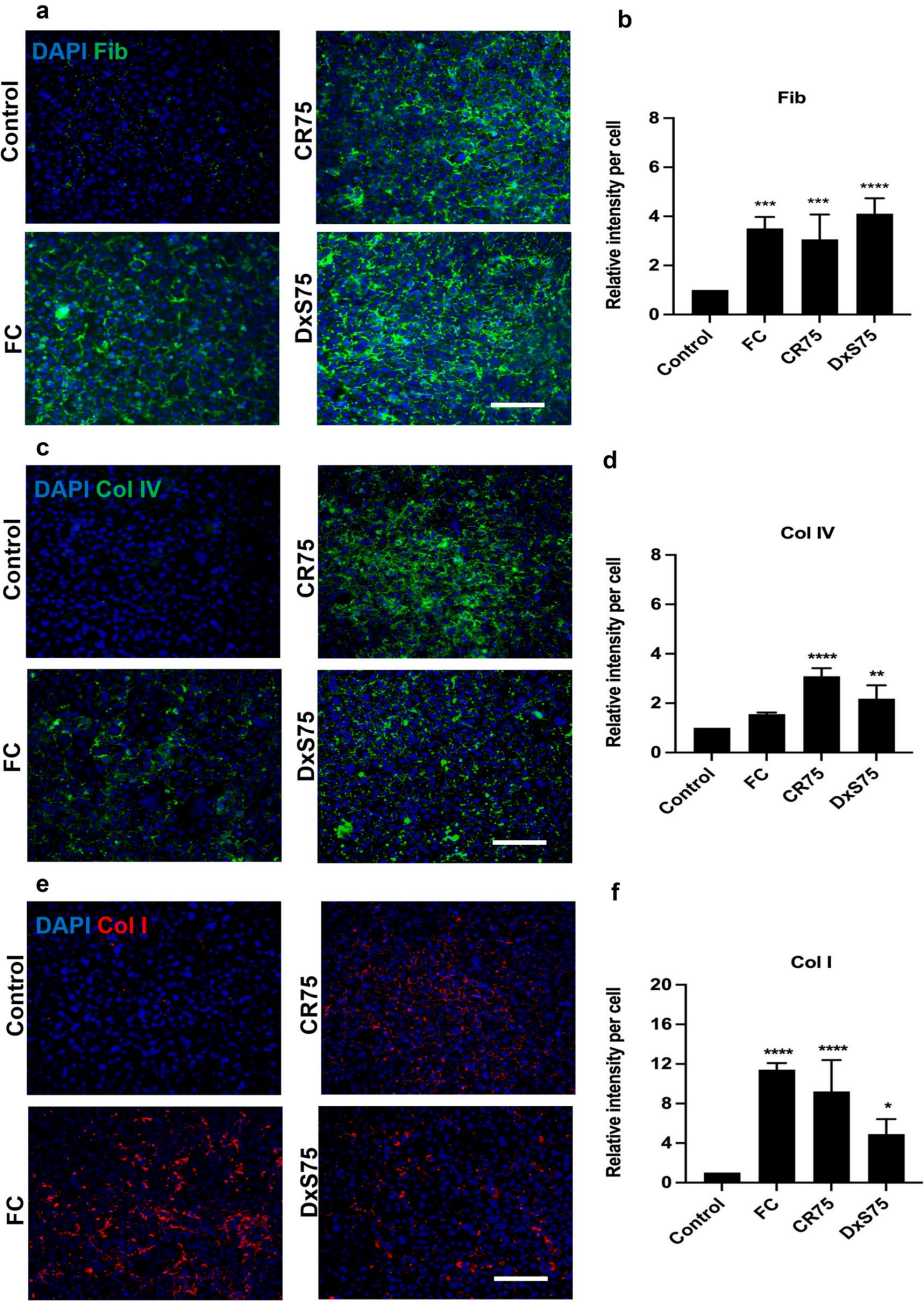
Effect of MMCs on fibrotic scar proteins in Neu7 astrocyte cultures. Representative immunofluorescent photomicrographs show fibronectin (Fib; **a**), collagen IV (Col IV, **c**) and collagen I (Col I; **e**) expression in Neu7 astrocytes without MMC (control) and with the MMCs FC, CR75 and DxS75. Bar charts show the relative fluorescence intensity per cell analysis of Fib (**b**), Col IV (**d**) and Col I (**f**). Scale bars = 100 µm. n = 3. Mean ± SD. *p = 0.02, **p = 0.001, *** p = 0.0004, **** p < 0.0001 compared to control. Tukey’s posthoc test using one-way ANOVA

**Fig. 8:**
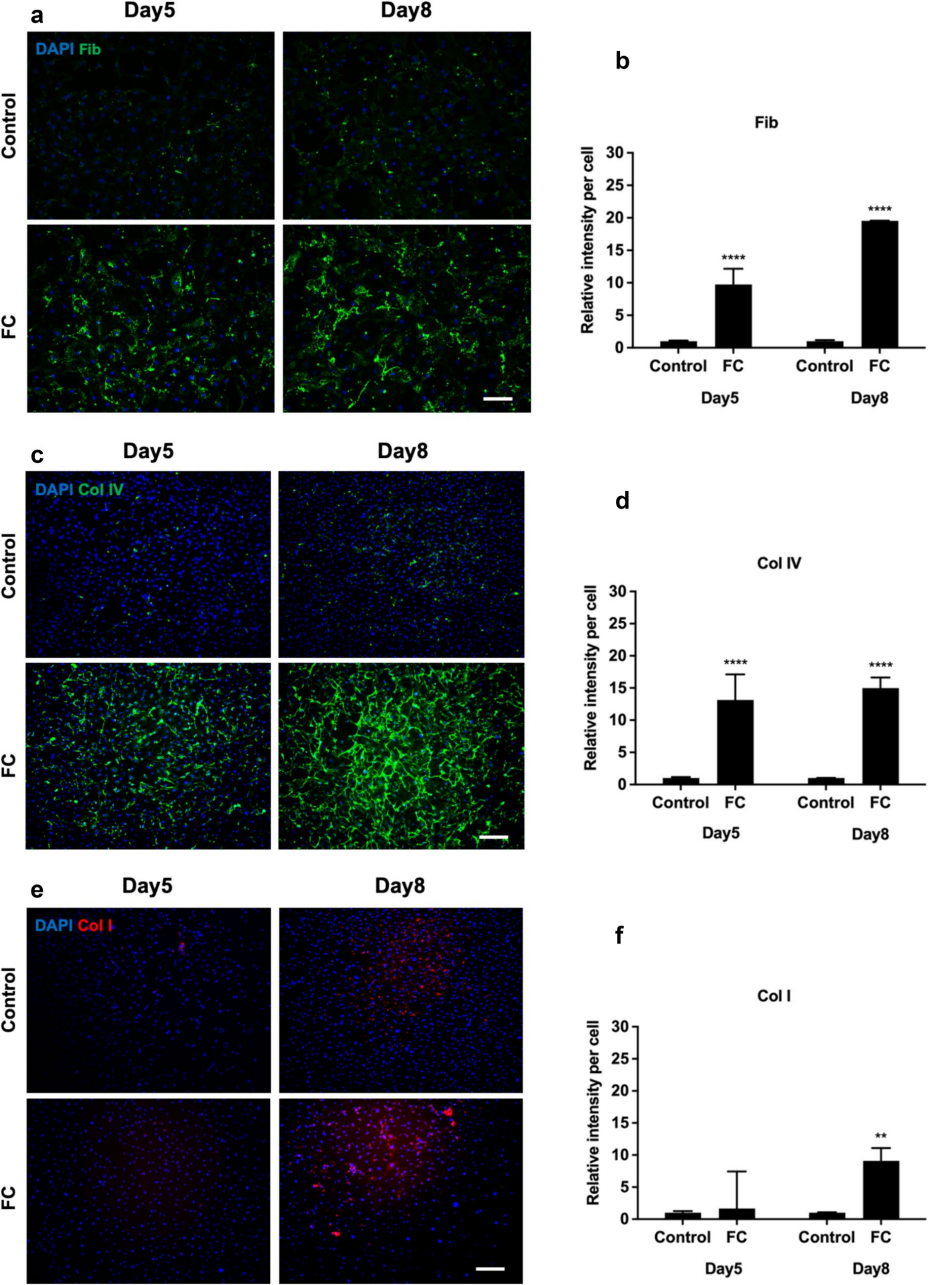
Effect of FC on fibrotic scar proteins in primary astrocyte cultures. Representative immunofluorescent photomicrographs show fibronectin (Fib; **a**), collagen IV (Col IV, **c**) and collagen I (Col I; **e**) expression in primary astrocytes on day 5 and day 8 without FC (Control) and with the MMC FC. Bar charts show the relative fluorescence intensity per cell analysis of Fib (**b**), Col IV (**d**) and Col I (**f**). Scale bars = 100 µm. n = 3. Mean ± SD. **p = 0.001, **** p < 0.0001 compared to control. Tukey’s posthoc test using one-way ANOVA

The deposition of collagen I, collagen IV, and fibronectin, which are key components of fibrotic scars after SCI, was significantly increased in the presence of MMC for both Neu7 and primary astrocytes compared to the control group (Figures. 7 and 8). The production of fibronectin by Neu7 astrocytes increased significantly on day 5 when FC, CR75 and DxS75 were added to the culture media (Fig. 7a, b). For collagen IV, the greatest deposition was observed with CR75 on day 5 (Fig. 7c, d). Among the different MMCs, the highest deposition of collagen I occurred in the presence of FC in Neu7 astrocytes (Fig. 7e, f). These findings suggest that different MMC agents can distinctly influence the deposition of specific ECM components by Neu7 astrocytes, thereby potentially affecting the formation and characteristics of fibrotic scars.

In primary astrocyte cultures, both fibronectin and collagen IV deposition increased in the FC-treated groups at both day 5 and 8 (Fig. 8a, b and 8c, d respectively). Although, there was no significant change in collagen I deposition observed on day 5 compared to the control group, by day 8 there was a significant increase in collagen I deposition in the FC-treated group (Fig. 8e, f).

The fibrotic scar located at the lesion core after SCI is characterized by the presence of fibroblasts. Fibrotic scars can impede functional recovery, posing a significant challenge to the regeneration and repair of the spinal cord (Ayazi et al. 2022). Moreover, astrocytes can deposit fibronectin, especially under pathological conditions such as neuroinflammation (Zhu et al. 2015). In response to injury or inflammatory stimuli like tumour necrosis factor-α treatment, astrocytes can synthesize and accumulate fibronectin within the ECM. This excessive fibronectin deposition can contribute to reactive astrogliosis and activate proinflammatory signalling pathways to produce nuclear factor-kappa B (Chu et al. 2023). In addition, astrocytes are integral to the production of ECM components including collagen IV (Wareham et al. 2024; Vogelaar et al. 2015).

Our findings are consistent with the observed impact of FC on the neuroglial ECM in vitro (Vo et al. 2022), where it was shown that FC significantly enhanced the deposition of key ECM proteins, including collagen IV, fibronectin and laminin in human-induced pluripotent stem cell derived astrocytes. This enhanced ECM deposition correlated with improved development of dopaminergic neuron networks, evidenced by increased neuronal extensions and synaptic activity, aligning with the reported physiological benefits. FC and CR also effectively boosted ECM accumulation in cultures of the glioblastoma cell line GL261. Specifically, the addition of CR or FC to the culture medium for one week significantly increased the total collagen and glycosaminoglycan content, as well as the immunoreactivity for fibronectin (Louisthelmy et al. 2023).

Astrocytes secrete and deposit a diverse array of proteoglycans and ECM proteins that influence their own growth and contribute to the development of neural microenvironments and networks (Sofroniew 2009). Analysis of ECM remodeling may allow for discovery of new therapeutic targets in relation to glial and fibrotic scar (Butt et al. 2023). Most existing knowledge on astrocyte-ECM production stems from studies focused on pathological processes, such as glial and fibrotic scar formation and SCI. The physiological ECM-building capabilities of astrocytes in vitro remain underexplored. To address this gap, we designed experiments incorporating MMC agents CR, DxS and FC to enhance ECM deposition in astrocyte monolayer cultures. While this technique has been successfully applied to mesenchymal cells (Zeiger et al. 2012), fibroblasts (Djalali-Cuevas et al. 2024) and epithelial cells (Tsiapalis and Zeugolis 2021; McLenachan et al. 2017), its application in glial cells remains largely unexplored. This study showed increased ECM protein deposition in both Neu7 cells and primary astrocytes in vitro after treatment with MMCs. However, FC proved to be more suitable for both cell types, as primary astrocytes did not respond well to treatment with CR and DxS.

## Conclusions

In conclusion, while Neu7 astrocytes were more adaptable to the presence of negatively charged molecules, CR and DxS significantly disrupted the morphology of primary astrocytes, highlighting the potential sensitivity of primary astrocytes to changes in their ionic environment. Primary astrocytes cultured with FC demonstrated an increase in expression of GFAP, CS56, fibronectin and collagen IV, all of which are key components found in SCI scarring. In contrast, Neu7 cells did not show an increase in collagen IV when treated with FC but they also tend to grow very rapidly, making them unsuitable for longer time points in studies. Therefore, primary astrocytes were more suitable for use in this in vitro model of SCI, as they not only accurately reflected the protein composition observed in actual SCI scars but also offered more stable growth over extended periods. This novel culture system opens up a new avenue for screening therapeutic compounds to modulate the environment of the glial and fibrotic scar in spinal cord repair. The model promotes the 3Rs in animal experimentation and allows the rapid formation of glial and fibrotic scars in vitro, a process which requires approximately two weeks within in vivo studies (Li et al. 2021; Zhang et al. 2022a, b). By targeting ECM remodeling, it may be possible to improve axonal repair and facilitate functional recovery after SCIs.

## Methods

### Cell culture

All cell culture media and reagents were purchased from Merck (Dublin, Ireland) unless otherwise stated. Primary astrocytes were purchased from Innoprot (Cat. No. P10202) and cultured in primary astrocyte culture medium provided by the company (Innoprot, Cat. No. P60101) consisting of Basal Medium supplemented with 5% foetal bovine serum (FBS), 1% astrocyte growth supplement (AGS), and 1% penicillin/streptomycin (P/S). Prior to cell seeding, flasks were coated with poly-L-lysine (PLL) (Cat. No. P8920) for 1 h at 37 °C. After coating, flasks were rinsed twice with Dulbecco’s phosphate-buffered saline (DPBS). The Neu7 astrocyte cell line, obtained from Professor James Fawcett (Cambridge, UK), was cultured in low glucose Dulbecco’s modified Eagle’s medium (DMEM, Cat. No. D6046) supplemented with 10% FBS (Cat. No. F7524), 1% P/S (Cat. No. P0781) and 1% L-glutamine (Cat. No. G7513). All cells were maintained in a cell culture incubator at 37 °C in a 5% humidified CO_2_ atmosphere.

### Cell treatment with MMCs

The MMCs employed in this study comprised CR (Cat. No. C1013), DxS (TdB Labs, Cat. No. DB051), and FC (Ficoll 70 kDa and Ficoll 400 kDa, Sigma Life Sciences, Cat. No. F2878, Cat. No. F4375, respectively). Prior to use, the MMCs underwent sterilization under UV light for 1 h and were subsequently dissolved in media. CR and DxS were dissolved in the respective cell-specific media at 1 mg/ml, while FC was dissolved at 37.5 mg/ml for 70 kDa and 25 mg/ml for 400 kDa. The concentration of FC was optimized in previous studies (Chen et al. 2009). However, to optimize the concentrations of CR and DxS, stock solutions were further diluted in the appropriate culture medium to concentrations of 25, 50, 75 and 100 μg/ml. Furthermore, 100 μM L-ascorbic acid 2-phosphate sesquimagnesium salt hydrate (Sigma-Aldrich, Cat. No. A8960) was freshly added to all MMC solutions at 1 μl/ml, as it is critical for collagen synthesis. The MMCs were introduced to cells 24 h after initial seeding at a density of 25,000 cells/cm^2^, with fresh media replenished every two days. Cell morphology was assessed using a Leica phase contrast microscope, and images were captured accordingly. Each experiment was carried out in triplicate after 5 and 8 days growth with MMCs to allow for separate biological replicates.

### Cell viability

Cell viability in the presence of the MMCs was assessed using the live/dead cell viability kit (Invitrogen, Cat. No. L3224). Cells were seeded onto 8-well chamber slides (Ibidi) at a density of 25,000 cells/cm^2^, and MMCs were added after 24 h. On day 5 and day 8, a live/dead assay solution was prepared by mixing calcein (1:1000) and ethidium bromide (1:500) with the appropriate culture medium. After rinsing the cells twice with DPBS, 200 μl of the live/dead solution was added and incubated with cells for 15–20 min at 37 °C. Cell viability was then evaluated by capturing images (in three replicate wells) of live/dead cells at 10 $\times$ magnification using an Olympus IX81 fluorescence microscope.

### Cell metabolic activity

The metabolic activity of cells in the presence of MMCs was evaluated using the alamarBlue assay (Invitrogen, Cat. No. A50101). Cells were initially seeded in a 24-well plate at a density of 25,000 cells/cm^2^. After 24 h, culture media containing MMCs was added (D2). On day 5 and day 8, the media was replaced with a solution consisting of alamarBlue reagent diluted at a ratio of 1:10 in culture medium. The cells were then incubated at 37 °C for 1–2 h. Subsequently, 25 μl of the incubated AlamarBlue solution and 75 μl of PBS were added to a black 96-well plate (Costar). The fluorescence was measured using a Hidex Sense microplate reader at wavelengths of 560/590 nm and percentage of cellular metabolic activity was estimated in three replicate wells. All measurements at both Day 5 and Day 8 were normalized to the Day 5 control group, allowing for consistent comparison across time points despite the reduced cell attachment at the later timepoint.

### Immunocytochemistry

Neu7 cells and primary astrocytes were seeded onto 8-well chamber slides at a density of 25,000 cells/cm^2^, with 200 μl of media per well. After 24 h, media containing MMCs were introduced to the cells. On day 5 and day 8, the cells were washed with PBS and fixed with 4% paraformaldehyde (PFA, Cat. No. 1.00496) for 15 min. Following fixation, the cells were blocked with 3% normal goat serum (NGS, Cat. No. G9023) for 10–15 min at room temperature. Primary antibodies (monoclonal mouse anti-glial fibrillary acidic protein (GFAP) (1:400, Dako, Cat. No. Z0334), polyclonal rabbit anti-fibronectin (1:200, Fisher Scientific, Cat. No. 16834864), polyclonal rabbit anti-collagen IV (1:200, Abcam, Cat. No. ab6586), polyclonal mouse anti-collagen I (1:200, Abcam, Cat. No. ab6308) and monoclonal mouse anti-CSPGs (1:200; CS56, Cat. No. C8038)) were prepared in antibody diluent solution (PBS containing 3% NGS and 0.02% Triton-X detergent). The cells were incubated with the primary antibody solution for 2 h at 37 °C. After three washes with PBS, secondary antibodies (anti-mouse Alexa Fluor 594 and anti-rabbit Alexa Fluor 488 (Invitrogen, Cat. No. A32742, Cat. No. A11008, respectively)) were diluted (1:200) in PBS and incubated with the cells for 30 min at room temperature. Subsequently, the cells were again washed three times with PBS and stained with DAPI (Thermo Fisher Scientific, Cat. No. D1306) (1 μg/ml) for 4 min at room temperature. Following three additional PBS washes, the cells were examined under an Olympus IX81 fluorescence microscope at 10 $\times$ and 20 $\times$ magnification in three replicate wells per antibody.

### Image analysis

All imaging was carried out using the same exposure time. Estimation of cell viability was carried out using ImageJ to count the number of live and dead cells per image (3 images per well) using the nuclear particle analyser to determine the percentage of live cells (% cell viability). The fluorescence intensity per cell of all immunocytochemical markers was estimated using ImageJ to examine the relative fluorescence per image (4 images per well), and this value was divided by the number of nuclei in each image (estimated using the nuclear particle counter). A negative control was performed to assess the background fluorescent signal, with any fluorescence signal above this background considered positive.

### Statistical analysis

Data is presented as mean ± standard deviation (SD) with experiments conducted in triplicates. Statistical analysis was performed using GraphPad Prism® software, employing one-way analysis of variance (ANOVA) and Tukey’s posthoc test for comparisons among groups. Statistical significance was considered at probability (p) ≤ 0.05.

## Supplementary Information

Below is the link to the electronic supplementary material.

Supplementary file1 (DOCX 2833 KB)

## Data Availability

No datasets were generated or analysed during the current study.

## Abbreviations

Col: Collagen
CR: Carrageenan
DxS: Dextran sulphate
ECM: Extracellular matrix
FC: FicollⓇ cocktail
Fib: Fibronectin
GFAP: Glial fibrillary acidic protein
MMC: Macromolecular crowding
SCI: Spinal cord injury
