# Supplementary material for: The effect of macromolecular crowders on deposition of extracellular matrix in astrocyte cultures
Source: Cell Tissue Res. 2025 May 19;401(2):129–43. doi: 10.1007/s00441-025-03980-4 (PMC12325430; doi:10.1007/s00441-025-03980-4)
Supplement: Supplementary file 1 — Supplementary file1 (DOCX 2833 KB) [file 441_2025_3980_MOESM1_ESM.docx]

**Supplementary figure 1**


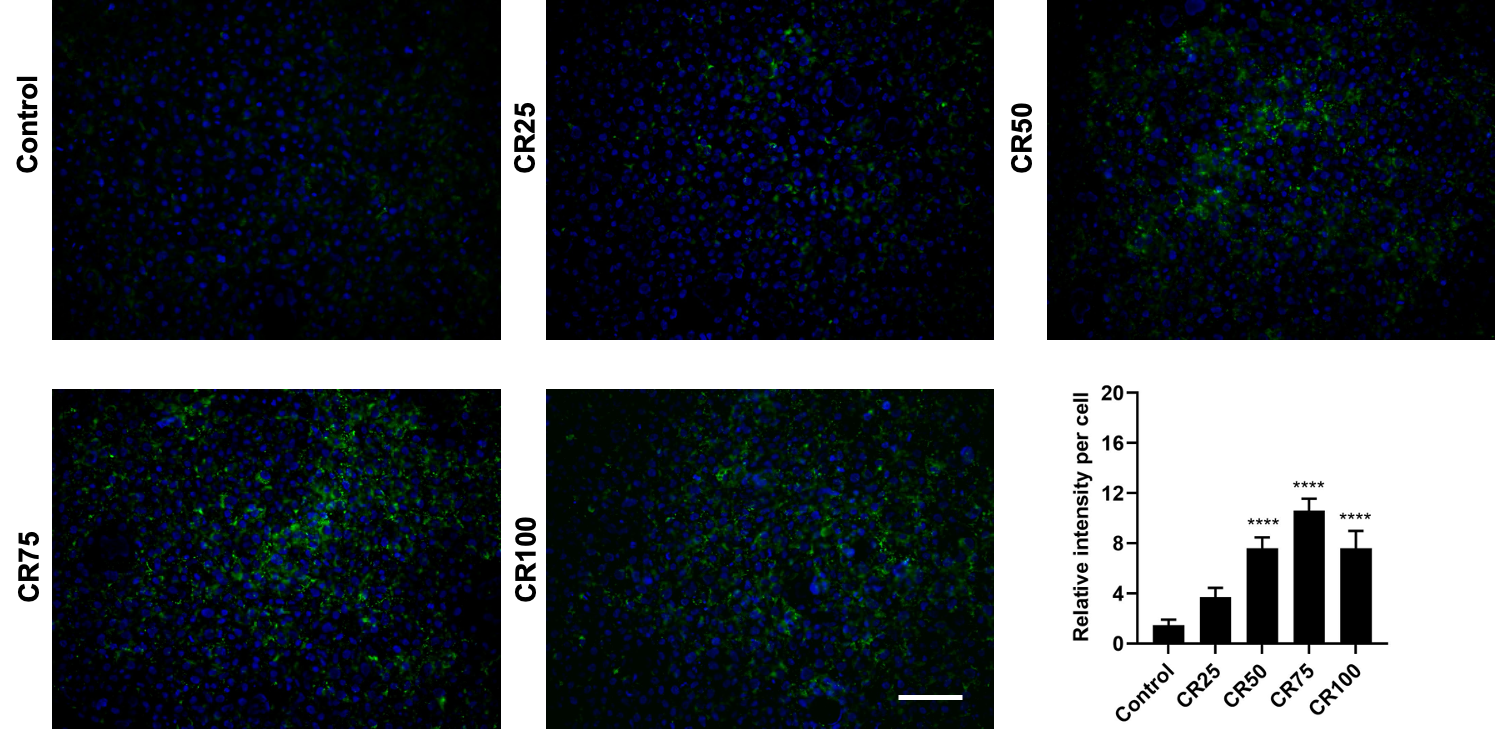


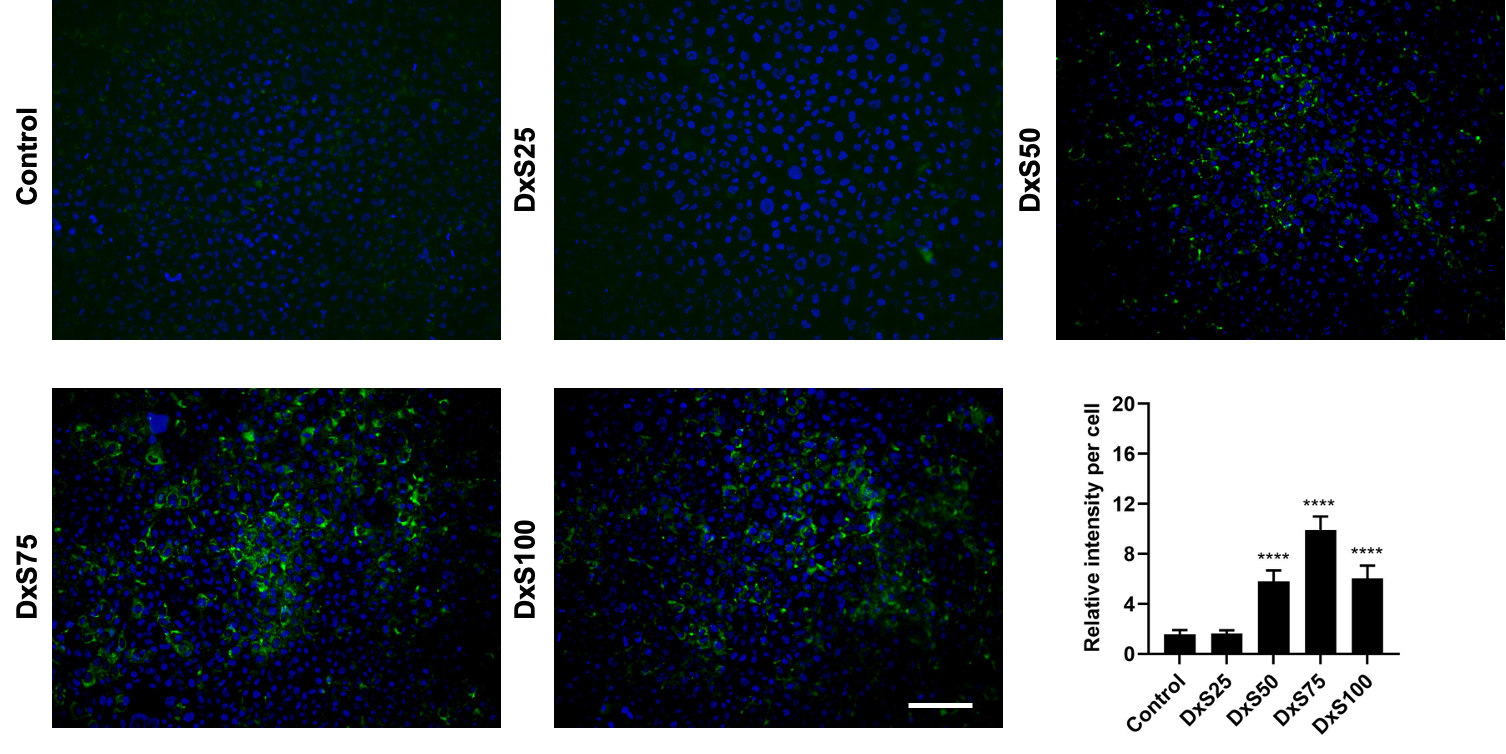


**Figure S1. Effect of MMCs on collagen I deposition in Neu7 astrocyte cultures. Representative immunofluorescent photomicrographs show collagen I (Col I) deposition in Neu7 astrocytes without MMC (Control) and with the MMCs CR and DxS at 25, 50, 75, and 100 μg/ml concentration. Blue, DAPI-stained nuclei; green, anti-collagen I antibody. Bar charts show the relative fluorescence intensity per cell analysis of Col I. Scale bars = 100 µm. n=3. Mean ± SD.**** p<0.0001 compared to control. Tukey’s posthoc test using one-way ANOVA.**
